# Probing the Relationship between Anti*-Pneumocystis carinii* Activity and DNA Binding of Bisamidines by Molecular Dynamics Simulations

**DOI:** 10.3390/molecules20045942

**Published:** 2015-04-03

**Authors:** Teresa Żołek, Dorota Maciejewska, Jerzy Żabiński, Paweł Kaźmierczak, Mateusz Rezler

**Affiliations:** Department of Organic Chemistry, Faculty of Pharmacy, Medical University of Warsaw, Banacha 1, Warsaw 02 097, Poland; E-Mails: tzolek@wum.edu.pl (T.Ż.); jzabinski@wum.edu.pl (J.Ż.); pawelkaz1@upcpoczta.pl (P.K.); matr@o2.pl (M.R.)

**Keywords:** water mediated DNA interaction, molecular docking, pentamidine analogs, anti-*Pneumocystis carinii* activity, DNA melting temperature

## Abstract

The anti-*Pneumocystis carinii* activity of 13 synthetic pentamidine analogs was analyzed. The experimental differences in melting points of DNA dodecamer 5'-(CGCGAATTCGCG)_2_-3' complexes (ΔT_m_), and in the biological activity measured using ATP bioluminescence assay (IC_50_) together with the theoretical free energy of DNA-ligand binding estimated by the proposed computational protocol, showed that the experimental activity of the tested pentamidines appeared to be due to the binding to the DNA minor groove with extended AT sequences. The effect of heteroatoms in the aliphatic linker, and the sulfonamide or methoxy substituents on the compound inducing changes in the interactions with the DNA minor groove was examined and was correlated with biological activity. In computational analysis, the explicit solvent approximation with the discrete water molecules was taken into account, and the role of water molecules in the DNA-ligand complexes was defined.

## 1. Introduction

The antimicrobial activity of aromatic bisamidines is well known, but only pentamidine is clinically used in the treatment of pneumonia caused by the opportunistic fungus *Pneumocystis jiroveci*, against antimony-resistant leishmaniasis, and initial stage human African trypanosomiasis [1,2,3,4,5,6,7,8,9]. Serious human health problems caused by these diseases in developing countries as well as after the spread of AIDS have stimulated research to find new drugs against them. There are several indications that pentamidine has a wider range of antimicrobial activity [10,11], however its high activity is associated with high toxicity and low bioavailability [12]. Recently we have found that some pentamidine analogs had little to no toxicity [13], and although they are not as quite potent as pentamidine, they hold promise for a decreased side effect within mammalian host.

The exact mechanism by which this drug acts *in vivo* is still under study and the molecular targets of pentamidine have not been unambiguously identified yet, but it is known that bisamidines accumulate in DNA-containing organelles and interfere with numerous cellular systems including ion channels as well as enzymes and modulate some RNA–protein, DNA–protein, and protein-protein interactions [14,15]. Furthermore, fluorescence microscopy results indicate that adenine-thymine sequences of mitochondrial kinetoplast DNA (kDNA) in trypanosomes are the primary targets for bisamidines which interfere with the replication of kDNA interacting with DNA in a nonintercalative manner in the adenine-thymine rich region (AT-rich) of the minor groove [16].

The molecular modeling studies of pentamidine analogs were reported and the free energies of binding with the DNA minor groove based on structural data obtained by X-ray diffraction measurements of pentamidine-oligonucleotide complexes [17,18,19,20,21] were used as a valuable tool to understand the structure-activity relationship, and for a design of new chemotherapeutics [20,21,22,23,24,25].

Since good correlation was found for many pentamidine analogs [26,27] between DNA binding affinity and stabilization of the DNA helix, estimated by melting temperature (T_m_), the design of amidine dications that target the minor groove of DNA has evolved as a productive concept for discovering new potent anti-*Pneumocystis* agents. Although the AT specific interactions are very informative, predicting the role of structural features that would be optimal for generating the biological effect is still worth the effort.

The investigations of shape complementarity between the minor groove DNA and the ligand are important for the biological activity analysis, but in some cases the DNA minor groove is able to form strong interactions even with compounds lacking the complementary shape. The water-mediated interactions are crucial for this recognition model promoting the flexibility required for the DNA binding [28]. Water molecules act as effective hydrogen bond donors or acceptors, and are able to form hydrogen bond networks. Their presence should be taken into account in the rational drug design process as an important component of biological systems.

In the present paper, the theoretically modeled system was built on the basis of the X-ray crystal structure of a complex between the dodecamer 5'-d(CGCGAATTCGCG)_2_-3' and pentamidine (RCSD PDB: 1D64.pdb) [17]. To take into account water-mediated interactions the energies of pentamidine analogs complexes with DNA were analyzed in the explicit system wherein the dodecamer, along with its associated 77 “crystal water molecules” were placed in a rectangular box of ~4000 TIP3P [29] waters. The majority of “crystal waters” occur in the first hydration shell of solvation and are involved in hydrogen bonding with the base functional groups, the oxygen atoms of the sugar groups, and the phosphodiester backbone of DNA [30].

To check the movements of DNA complexes during molecular dynamic simulations we tested the evolution of the structure of pentamidine and the DNA dodecamer 5'-(CGCGAATTCGCG)_2_-3' complex using the root-mean-square deviation (RMSD) analysis (see Supplementary Materials).

Thirteen pentamidine analogs **1**–**13** were selected to form a set of compounds to be tested (see Table 1 where their structures are presented) for which the *in vitro* ATP bioluminescent assay evaluating the activity against *Pneumocystis carinii* was published by us [13]. They are linear bisamidines with different heteroatoms or sulfonyl groups in the linker, and with methoxy groups at the benzene rings.

In the present paper, we examined whether or not the changes in activity (expressed in IC_50_) can be explained by the interactions with DNA in the AT-rich region of the minor groove. Another parameter which was considered in the investigations was the difference between the melting temperature of free DNA and DNA bonded with the tested compounds, which provides a good measure for ranking the compounds against DNA binding affinities (the values of ΔT_m_ were determined as shown in Experimental Section).

The purpose of this work was not only to provide useful information in order to better understand the DNA-binding properties of the linear bisamidine molecules, but also to find an effective tool for the future selection of bisamidines in pharmaceutical projects by utilizing the unique features of the DNA-bisamidine complexes defined during the theoretical simulations.

## 2. Results and Discussion

### 2.1. Relative DNA Binding Affinity: Changes of DNA Melting Temperature T_m_

Interactions with nucleic acid have been implicated for many aromatic bisamidines, and the evidence suggests that minor groove binding is at least in part responsible for their biological activity [3,16,17,18,19,20,21,22,23,24,25]. The measured, as indicated in Experimental Section, T_m_ ± SDT_m_ and ΔT_m_ values for compounds **1**–**13** are presented in Table 1 next to the chemical formulas shown for clarity. Compounds **1**–**13** displayed ΔT_m_ values in the range from 5.84 to 0.10 °C reflecting from stronger to much lower affinities to DNA than pentamidine. The highest increase of T_m_ was observed for compounds **5** and **10**, in which the middle CH_2_ group in the pentamidine molecule was replaced by O (compound **5**) or S (compound **10**) atoms, and additionally the phenolic O atoms are replaced by N-H groups (compound **5**). The compounds with the central methylene group replaced with N-CH_3_ or sulfonamide substituents did not increase the stability of DNA complexes. The least potent compounds in this series were those substituted with methoxy groups at benzene rings, and were characterized by the smallest values of ΔT_m_. These observations demonstrated that anti-*Pneumocystis* activity of the linear pentamidine analogs was associated with their ability to bind to the DNA dodecamer 5'-d(CGCGAATTCGCG)_2_-3'.

**Table 1:** The structures of pentamidine analogs **1**–**13**, an increase of melting temperature of the 5'-(CGCGAATTCGCG)_2_-3' dodecamer complex after complexation (ΔT_m_), anti-*Pnemocystis carinii* activities (IC_50_), and free energies of binding (ΔG_bind_) of DNA-ligand using MM-PBSA method.

Name	Chemical Structure	(T_m_ ± SDT_m_) [°C]	ΔT_m_ [°C]	(IC_50_ ± SDIC_50_) [µM]	ΔG_bind_ [kcal/mol]
**1**	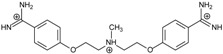	56.41 ± 0.23	3.12	1.73 ± 0.93	−35.5
**2**	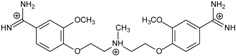	55.41 ± 0.62	2.12	3.41 ± 2.00	−25.6
**3**	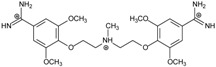	54.76 ± 0.42	1.47	6.26 ± 2.57	−20.2
**4**	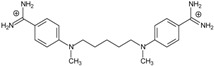	54.46 ± 0.22	1.17	1.99 ± 1.00	−19.9
**5**	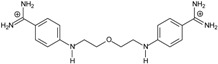	59.13 ± 0.06	5.84	1.11 ± 0.21	−47.3
**6**	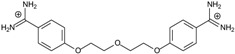	56.21 ± 0.28	2.92	2.13 ± 0.71	−26.9
**7**	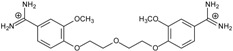	55.11 ± 0.83	1.82	4.21 ± 3.47	−22.0
**8**	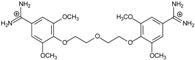	53.39 ± 0.85	0.10	12.99 ± 2.83	−10.7
**9**	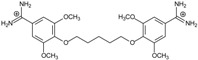	55.64 ± 0.72	2.35	3.00 ± 0.66	−27.2
**10**	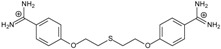	58.11 ± 0.22	4.83	1.18 ± 0.09	−44.3
**11**	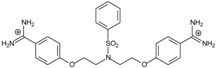	56.54 ± 0.62	3.25	1.33 ± 0.20	−39.7
**12**	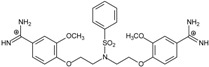	55.74 ± 0.72	2.45	2.66 ± 1.21	−27.3
**13**	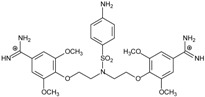	55.16 ± 0.81	1.87	4.16 ± 1.13	−22.4
**PN**	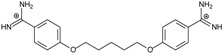	57.94 ± 0.21	4.65	0.51 [31]	−42.7

### 2.2. Relationship between the DNA Free Energy of Binding ΔG_bind_ and ΔT_m_

**Figure 1:**
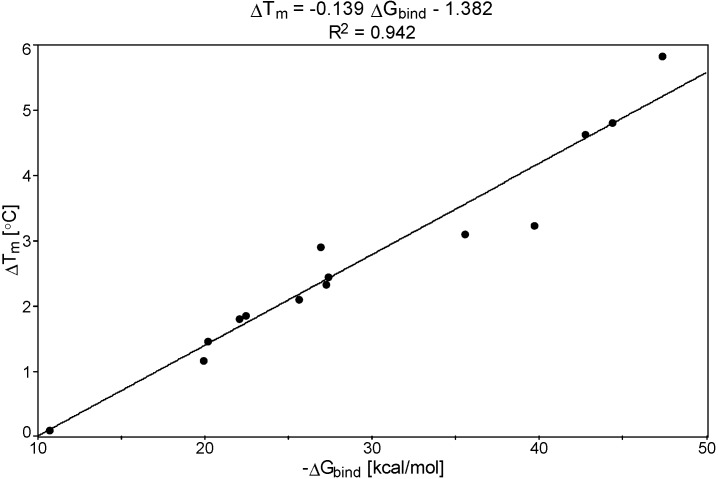
The plot of ΔT_m_ *versus* theoretically estimated DNA free energy of binding (−ΔG_bind_).

The values of computed total free energy of binding (−ΔG_bind_), determined as shown in Experimental Section (Section 3.5), are presented in Table 1. Free energy values for each component of complexes are given in Table S1 (see Supplementary Materials). A linear correlation was found between the experimental differences of DNA melting temperature (ΔT_m_) and the theoretically estimated binding free energies of the DNA-bisamidine complexes (−ΔG_bind_). Figure 1 shows the graphical relationship together with the linear equation fitted to the data: ΔT_m_ = −0.139ΔG_bind_ − 1.382. The correlation coefficient R^2^ for this equation was high, and was equal to 0.942.

Higher interaction energies were observed for higher melting points differences: the ligands with the lowest ΔT_m_ < 2.5 °C (**2**, **3**, **4**, **7**, **8**, **9**, **12** and **13**) were represented by ΔG_bind_ values between −19.9 kcal/mol to −27.3 kcal/mol, the ligands with moderate ΔT_m_ values between 3.0 and 3.5 °C (**1**, **6**, and **11**) were represented by ΔG_bind_ values in the range from −26.9 kcal/mol to −39.7 kcal/mol, and the ligands with the highest ΔT_m_ (**5**, **10**; ΔT_m_ > 4 °C) have the highest ΔG_bind_ values (~ −44.0 to 47.0 kcal/mol). From the energy and ΔT_m_ analyses it can be seen that small structural modifications had a profound influence on both parameters.

The compounds which weakly interact with DNA (**2**, **3**, **4**, **8**, **12** and **13**) have simultaneously the N atoms replacing the methylene group or the O atoms in the connecting chain and/or two or four methoxy groups at benzene rings. Structurally similar compounds (with the O or N heteroatoms, and *N*-sulfobenzene substituent in the middle of the linker) but without methoxy groups (**1**, **6** and **11**) interacted quite significantly with DNA. The introduction of aromatic sulfobenzene substituents at a central N atom did not increase the interactions with the DNA fragment (compounds: **7**, **9**). The most potent compounds **5** and **10** have three heteroatoms in the aliphatic linker: the O or S atoms in the middle and the N or O atoms at the end of aliphatic chains.

The strength of intermolecular interactions of all ligands in the minor groove of DNA dodecamer 5'-d(CGCGAATTCGCG)_2_-3' reflected well the changes in the melting temperature of DNA, and can be taken into account in order to understand their DNA affinity.

### 2.3. Relationship between IC_50_ vs. ΔG_bind_ and ΔT_m_

**Figure 2:**
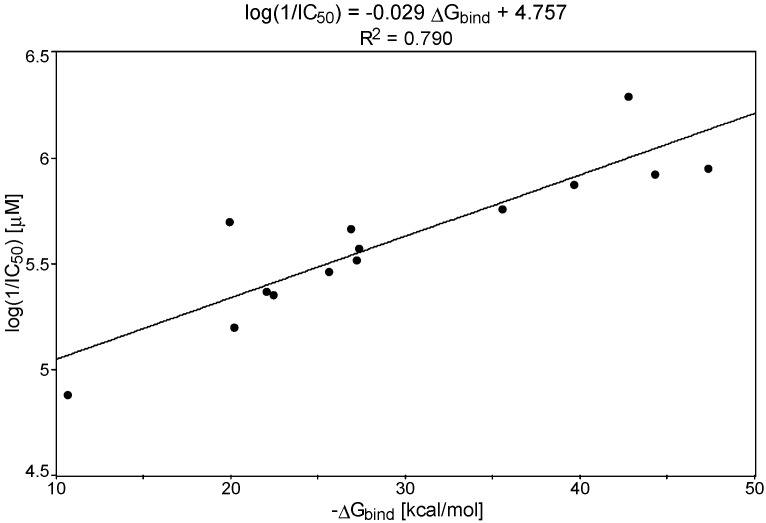
The plot of log(1/IC_50_) *versus* theoretically estimated DNA free energy of binding (−ΔG_bind_).

**Figure 3:**
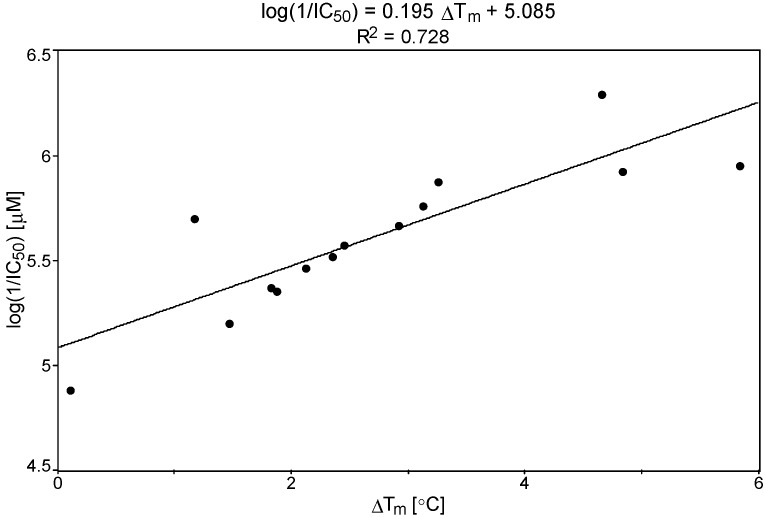
The plot of log(1/IC_50_) *versus* ΔT_m_ for the 5'-(CGCGAATTCGCG)_2_-3' dodecamer-bisamidine complexes.

The efficiency against *P. carinii* evaluated in the ATP bioassay for compounds **1**–**13** prompted us to analyze the relationships between IC_50_ and DNA interactions obtained from the T_m_ measurements and MD analysis of ligand-DNA complexes. In the tested series of pentamidine analogs, the activity expressed as log(1/IC_50_) showed linear correlations with free energy of binding ΔG_bind_ and ΔT_m_ values. Figure 2 and Figure 3 present the results.

The obtained equation for log(1/IC_50_) *vs.* (ΔG_bind_) was log(1/IC_50_) = −0.029ΔG_bind_ + 4.757 (R^2^ = 0.790), and for log(1/IC_50_) *vs.* ΔT_m_, was log(1/IC_50_) = 0.195ΔT_m_ + 5.084 (R^2^ = 0.728). These correlations (the increase in ΔG_bind_ values in connection with the increase in ΔT_m_ values and the increase of anti-*P. carinii* activity) supported the importance of the DNA interaction step in the mechanism of biological activity of those closely related pentamidine analogs.

The activity of reference compound-pentamidine **PN** defined by these models was the high but not the highest. It should be emphasized, that experimental activity is dependent on a drug uptake, but this parameter was not characterized by the models. However, the results of the T_m_ measurements support the DNA as the biological target of bisamidines, and can explain the correlation of DNA binding with biological activity.

### 2.4. Evaluation of Theoretical Models

Five compounds were used to evaluate the theoretical models (see Table 2). Three *N,N'*-alkyl-1, *n*-diylbis(4-amidinobenzamide) dihydrochlorides **TC1**–**TC3** were used as testing compounds because they include the bisamidine linker. It was interesting to check whether or not the proposed linear correlations could predict the lower anti*-P. carinii* activities observed for these pentamidine analogs [13].

**Table 2:** Evaluation of models: testing compounds **TC1**–**TC5**; experimental and theoretical parameters: ΔT_m_, IC_50_ and ΔG_bind_.

Name	Chemical Structure	(T_m_ ± SDT_m_) [°C]	ΔT_m_ [°C]	(IC_50_ ± SDIC_50_) [µM]	ΔG_bind_ [kcal/mol]
**TC1**	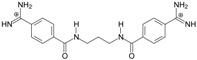	55.66 ± 0.22	2.37 Predicted: 2.31	2.99 ± 1.76 Predicted: 3.97	−26.6
**TC2**	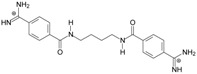	-	-	3.58 ± 0.02 Predicted: 4.65	−21.1
**TC3**	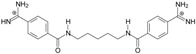	50.21 ± 0.71	−3.08 Predicted: −2.46	14.33 ± 0.42 Predicted: 10.75	7.7
**TC4**	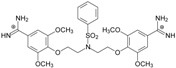	-	-	4.40 ± 1.39 Predicted: 4.93	−19.2
**TC5**	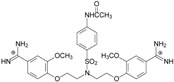	-	-	3.71 ± 1.19 Predicted: 4.37	−23.3

Next two **TC4** and **TC5** have the additional aromatic ring. Only compounds **TC1** and **TC3** were soluble enough for the melting temperature measurements, and were used for evaluation of two correlations: ΔT_m_ = f(−ΔG_bind_) and log(1/IC_50_) = f(−ΔG_bind_). The other compounds were used only for evaluation of the correlation log(1/IC_50_) = f(−ΔG_bind_). The predicted and experimental IC_50_ and ΔT_m_ values are given in Table 2. Clearly, both equations derived here have a predictive ability—the experimental and the computed values are close to each other.

### 2.5. Intermolecular Interactions of Ligands in DNA Minor Groove

**Figure 4:**
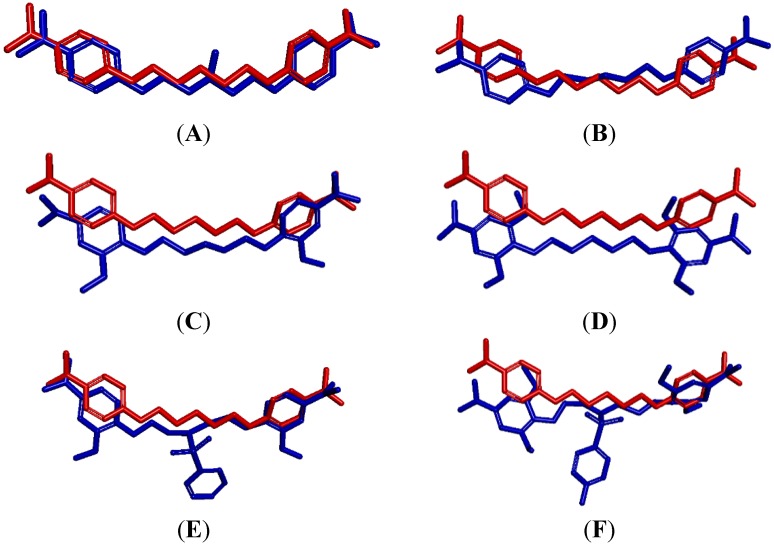
Illustration of conformational differences between pentamidine and its analogs in the DNA minor groove. Pentamidine is shown in red, and the ligands are shown in blue. (**A**) compound **1**; (**B**) compound **6**; (**C**) compound **7**; (**D**) compound **9**; (**E**) compound **12**; (**F**) compound **13**.

**Figure 5:**
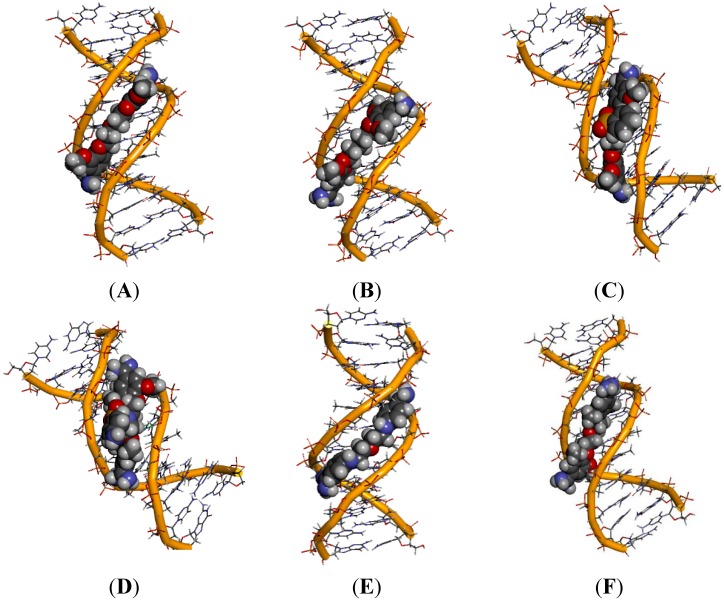
Location of ligands in the minor groove. (**A**) deformation of DNA by **7**; (**B**) deformation of DNA by **9**; (**C**) deformation of DNA by **12**; (**D**) deformation of DNA by **13**; (**E**) fitted conformation of **5**; (**F**) deformation of DNA by **8**.

To compare the impact of different substituents on anti-*P. carinii* activities with DNA binding profiles of **1**–**13**, the conformations of the tested bisamidines were analyzed in the A-T region of the dodecanucleotide minor groove in a position closely similar to that observed for pentamidine. Table S2 (see Supplementary Materials) presents the length of intermolecular distances which are responsible for key interaction with the DNA minor groove. Figure 4 shows the overlay of the ligands conformations (blue) on the pentamidine structure (red) to illustrate the position of the ligands within the DNA minor grove with respect to pentamidine. Figure 5 displays the location of the ligands in the DNA dodecamer 5'-d(CGCGAATTCGCG)_2_-3'.

The compounds **1**, **4**, **6** evidenced the effect of the N and O atoms in the aliphatic linker on the activity against *P. carinii*. They are quite potent and their IC_50_s are equal to 1.73, 1.99 and 2.13 µM, respectively. In these compounds, the O atoms at the end of the linker do not interact with DNA (as they do not interact in pentamidine) as well as the N and O atoms in the middle of the joining chain. The NCH_3_ group of **1**, which penetrates deeply into the minor groove of the DNA, interferes in the formation of hydrogen bonding. Only the amidinium groups are involved in the network of intermolecular interactions. In the DNA–compound **1** complex both amidinium groups are involved in seven direct and indirect hydrogen bondings with the DNA bases (adenine 6, adenine 5, adenine 18, cytosine 9), the O4' atom of 2'-deoxyribose molecules, and the O3' atom of phosphate groups.

In addition, water-mediated hydrogen bonds occur with the O2 atom of cytosine 9 and the O3' atom of phosphate groups at thymine 7. Compound **4**, in which two O atoms of the pentamidine linker are replaced by the NCH_3_ groups, interacts with two strands of DNA. We can observe three direct hydrogen bond contacts between the H atoms of amidine groups: the O4' atom of 2'-deoxyribose at adenine 18, the O3' atom of the phosphate group at adenine 6, and with the N3 atom of adenine 17.

In the DNA-compound **6** complex only two strong interactions are present: between the H atoms of the amidinium group and the O4' atom of the 2'-deoxyribose at adenine 18 and the O3' atom of the phosphate group at cytosine 9. Both compounds **1** and **6** occupy the same space in the DNA minor groove as pentamidine (Figure 4A,B).

The overall stability of DNA complexes changes dramatically for pentamidine analogs which have four methoxy groups at the benzene rings (**3**, **8**, **9**, and **13**). Two methoxy groups at the benzene rings had less pronounced consequence (**2**, **7**, **12**). The presence of these groups weakened the interactions between the H atoms of the amidinium groups and DNA atoms or destroyed them (see Supplementary Table S2).

The conformations of compounds **7** and **9** with two or four methoxy groups did not overlay with pentamidine (Figure 4C,D), and did not fit the DNA minor groove (Figure 5A,B). The methoxy groups are located out of DNA curvature causing deformations of the minor groove walls. The sulfonamide substituents hindered the adoption of pentamidine-like conformation by compounds **12** and **13** (Figure 4E,F), and are directed toward the mouth of the groove (Figure 5C,D).

In these DNA complexes, water molecules do not accumulate over the minor groove, which prevents them from the formation of water ribbons. Figure 5E,F present the location of compounds **5** and **8**, the best and the worst DNA fitting compounds, respectively.

The representative compounds were selected for a more detailed analysis. Compounds **5** and **10** were chosen as the most potent structures, compound **11** as an example of compounds with the additional aromatic ring, and compound **8** as the least potent structure bearing OCH_3_ substituents.

The most potent compound is **5** (its IC_50_ is equal to 1.11 µM) and it also shows the strongest DNA binding affinity. Its amidinium groups form a network of interactions (Supplementary Table S2). The strongest hydrogen bonds are between the O4' atom of 2'-deoxyribose oxygen atoms at adenine 6 and at adenine 18 (2.29, 2.35 Å long, respectively). The next interactions are at cytosine 9 and at cytosine 21 (3.07, 3.05 Å long, respectively). The N3 atoms of adenine 5 and adenine 18 also interact directly with amidinium groups (3.33 and 3.26 Å long, respectively). Moreover, compound **5** creates hydrogen bonds with the O3' atom of the phosphate group at cytosine 21 and at cytosine 9 (2.95 and 3.05 Å long, respectively). The water networks of compound **5** will be discussed later on.

A very potent compound **10** (with IC_50_ equal to 1.18 µM) also shows strong affinity to DNA. The linker joining benzamidine groups in **10** is engaged in intermolecular interactions. The S atom in the aliphatic chain creates interactions with the O2 atom of thymine 7 (2.95 Å long) allowing for location of compound **10** near the bottom of the DNA minor groove. Additional hydrogen bonds appeared between the phenolic O atoms and the O4' atom of 2'-deoxyribose at thymine 19. The direct hydrogen bonds contacts are also between the H atoms of amidine groups and the O4' atom of 2'-deoxyribose molecules at cytosine 9, adenine 18 and adenine 6 (3.00, 3.04 and 2.79 Å long, respectively) and with the O3' atom of the phosphate group at guanine 10 (3.15 Å long). Both the most potent compounds, **5** and **10**, have a concave shape, which allows them to match the curvature of the helical minor groove and slide deeply into the minor groove (Figure 5E).

Compound **11** belongs to the next group of the tested bisamidines which have the N atom with the sulfobenzene substituent in the middle of the aliphatic linker. It is quite potent (IC_50_ is equal to 1.33 µM) and its potency can be explained in terms of hydrogen bonding interactions with DNA. The ligand **11** occupies a symmetric position along the DNA minor groove. The one amidinium group forms the hydrogen bond with the O4' atom of 2'-deoxyribose molecules at guanine 10 and adenine 6 (3.09 and 3.14 Å long, respectively) and with the O3' atom of the phosphate group at cytosine 9 (2.26 Å long). There are also hydrogen bonds between the O2 atom of cytosine 21 and cytosine 9 (3.08 and 2.97 Å long, respectively).

The least potent compound **8** bearing four methoxy groups interacts with only one strand of DNA. We can detect only one direct hydrogen bond contact between the amidine hydrogen atom and the O3' atom of the phosphate group at cytosine 9 (2.84 Å long). This interaction with a single DNA strand caused the deformations of the minor groove as shown in Figure 5F, and decreased the free energy of binding ΔG_bind_. The introduction of four methoxy groups at the benzene rings forced a completely extended trans conformation of the aliphatic linker, and prevented the ligand from matching the DNA helix curvature. This can be the explanation of the mode of action of the methoxy groups, which also explains the weakened DNA binding affinity and the reduced activity against *P. carinii*.

### 2.6. Hydration at the DNA-Ligand Complexes

**Figure 6:**
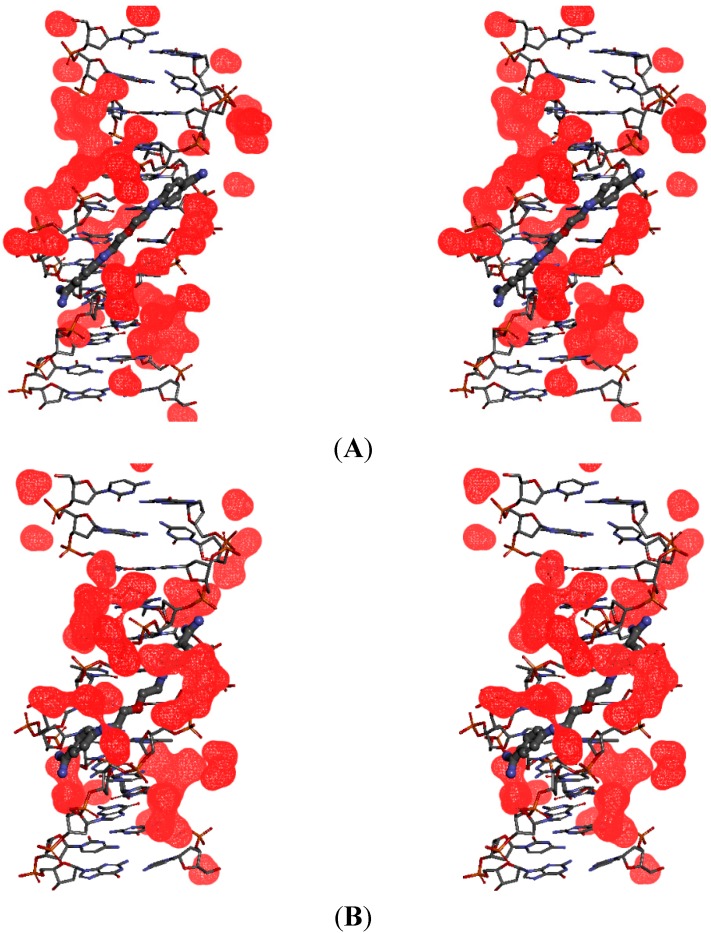
Stereo (cross-eye) views of the hydration surface (water density in red) associated with the movement of compound **5** within the 5'-(CGCGAATTCGCG)_2_-3' dodecamer. (**A**) In the time frames between 1.5 and 2 ns MD–there are no direct H-bond contacts between the DNA bases and both amidinium groups; (**B**) In the time frames between 5 and 10 ns MD–both amidinium groups in ligand make direct H-bond contacts with the DNA bases.

To characterize an active role of water molecules in the ligand binding with the minor-groove in the AT-rich binding site, some hydration sites which exhibit long nanosecond-scale residence time were analyzed using an approach proposed in papers [32,33]. This hydration network was observed as running along the length of the DNA dodecamer 5'-d(CGCGAATTCGCG)_2_-3', and the ligands as moving along the binding site during MD simulations. The average electron densities were generated for the water molecules located within 3.5 Å from any of the DNA atoms. To illustrate this phenomenon, the hydration surfaces were showed (Figure 6) as stereo views for two times frames: 1.5 and 2 ns (Figure 6A) and between 5 and 10 ns (Figure 6B) for the DNA complex of compound **5**.

We can see that in the first time frame the upper amidinium group (in the illustration) directly interacts with the floor of the minor groove through hydrogen bonds, but the bottom amidinium group makes contacts with the minor groove bases through water molecules (Figure 6A). In the second time frame both amidinium groups form water bridges which stretch the molecule and help in the DNA-ligand complex stabilization (Figure 6B).

**Figure 7:**
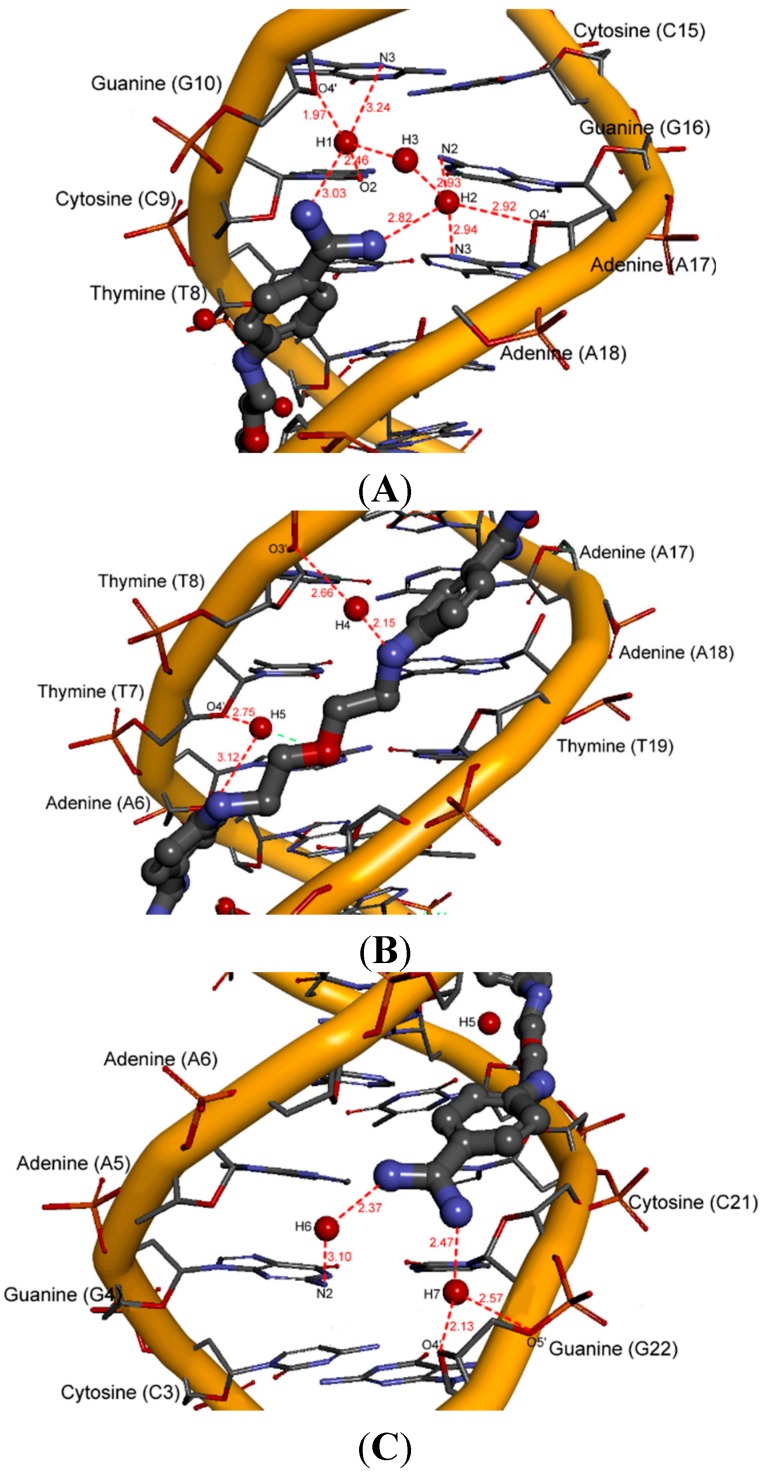
A view of hydration sites for compound **5** in the minor groove (red lines—hydrogen bonds; length [Å]). (**A**) hydration sites H1–H3; (**B**) hydration sites H4–H5; (**C**) hydration sites H6–H7.

To show the role of water molecules in the interaction of the ligands with the AATT binding site in detail, seven significantly ordered hydration sites denoted as H1–H7 were selected [32]. Three sites H1, H2 and H3 are located in the proximity of the upper amidinium group. Two sites H4 and H5 are located along the joining chain, and the last two, H6 and H7, are located in the proximity of the bottom amidinium group (see Figure 7A–C and Supplementary Table S2 in Supplementary Materials where those hydration sites are shown for compound **5**).

We decided to begin the description of the water network with compound **5** as the most potent substance (Figure 7) which formed all seven hydration sites. We can see the direct hydration site H1 present near the O2 atom of cytosine 9, the N3 atom of guanine 10 and the O4' atom of 2'-deoxyribose. The hydration site H2 is formed with participation of atoms: N3 (adenine 17), N2 (guanine 16) and O4' of 2'-deoxyribose (at adenine 17).

When sites H1 and H2 are occupied, the other water molecules form the hydration site H3 interconnecting sites H1 and H2. The hydration sites H4 and H5 are formed by the amine N atoms of the aliphatic chain and are located on one side of the linker. The last two hydration sites H6 and H7 are created thanks to the orientation of the amidinium groups towards the bottom of the minor groove, which makes possible additional contacts with the NH_2_ amidinium groups. The hydration site H6 is revealed as a water bridge at the N2 atom (guanine 4), whereas the hydration site H7 is present near the O4' atom of 2'-deoxyribose molecules at guanine 22. All the N atoms in compound **5** (two in the aliphatic linker and four in amidinium groups) that are hydrogen-bonded through the water molecules, are the points of water ribbons. 

**Figure 8:**
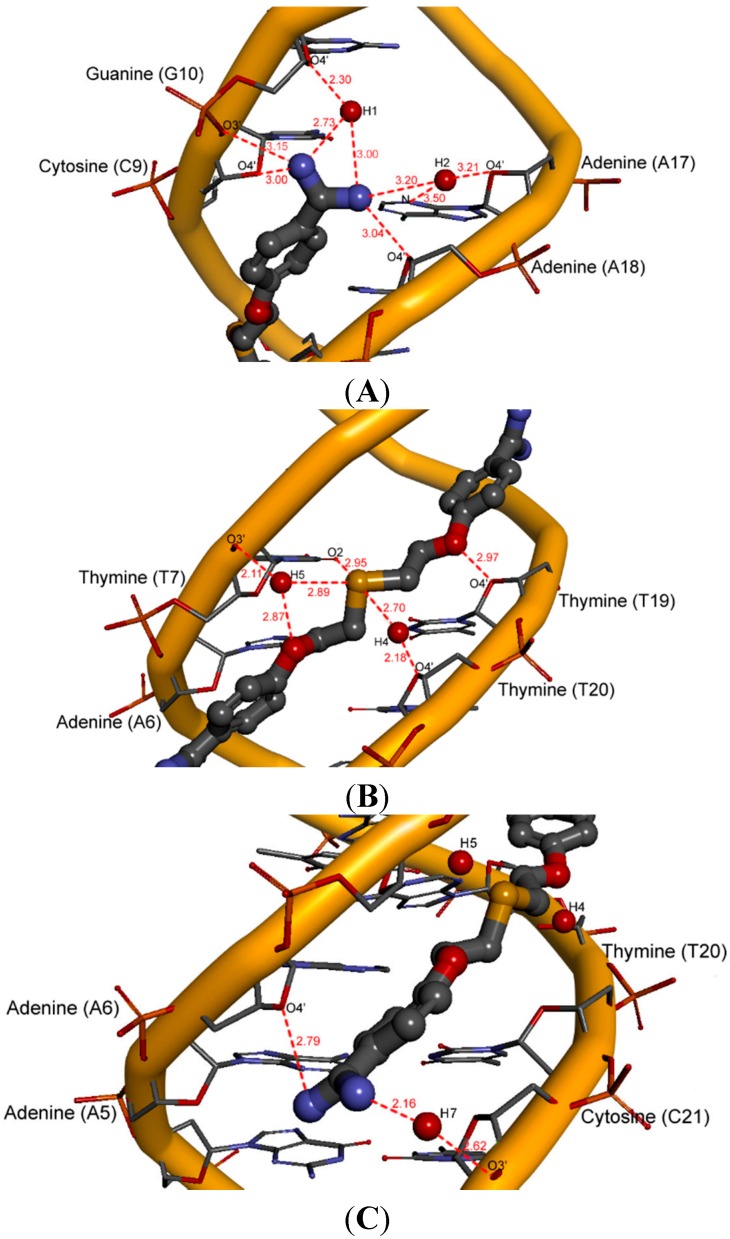
A view of five hydration sites for compound **10** in the minor groove (red lines—hydrogen bonds; length [Å]). (**A**) hydration sites H1–H2; (**B**) hydration sites H4–H5; (**C**) hydration site H7.

For only slightly less active compound **10** merely five hydration sites H1–H2, H4–H5 and H7 were observed (Figure 8).

We could see the direct hydration site H1 present near the O4' atom of 2'-deoxyribose at guanine 10 and the hydration site H2 formed with participation of the N3 atom (adenine 17) and the O4' atom of 2'-deoxyribose at adenine 17. The hydration site H4 is formed by the O4' atom of 2'-deoxyribose molecules at thymine 20, and the hydration site H5 is revealed as a water bridge between the O and S atoms and the O3' of the phosphate group at thymine 7. Both sites are located on opposite sides of the linker. The bottom amidinium group forms only one hydration site H7 between the O3' phosphate group at cytosine 21.

**Figure 9:**
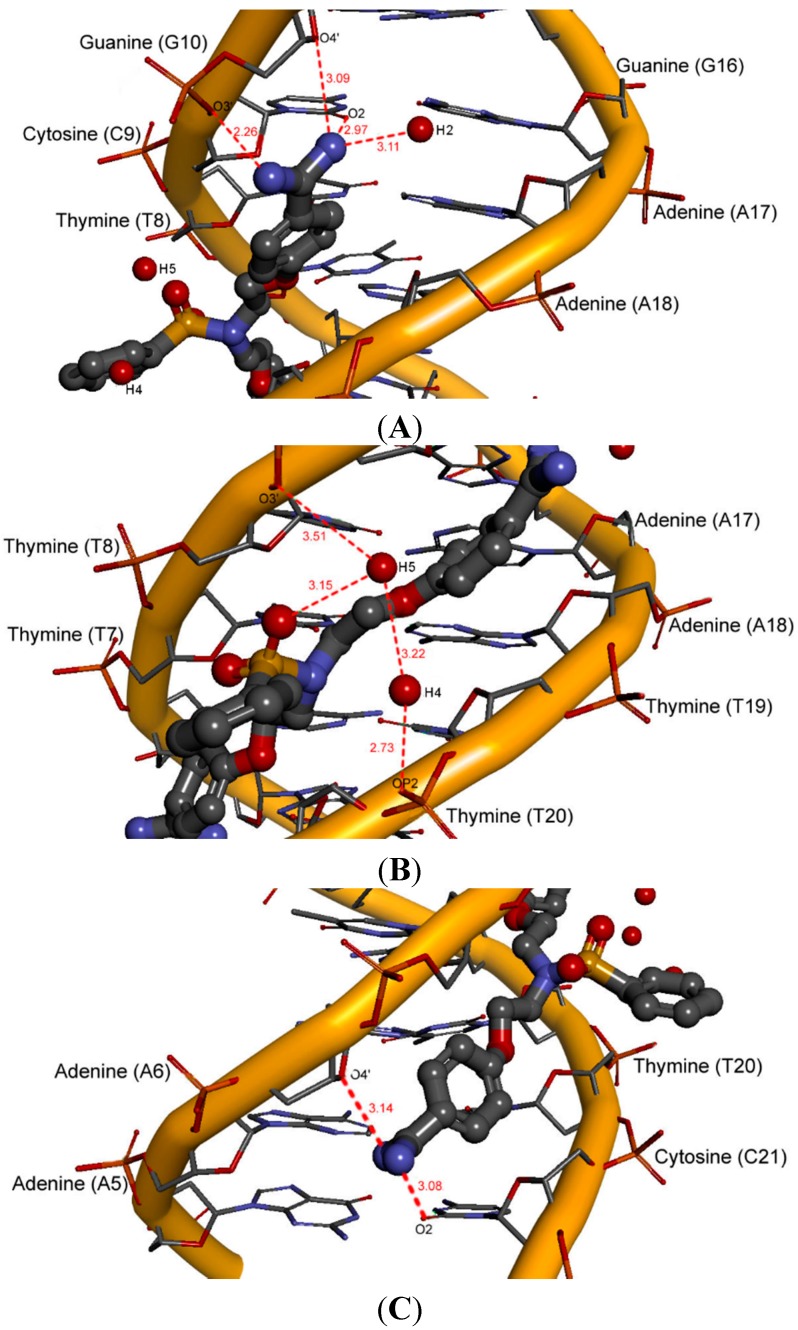
A view of three hydration sites for compound **11** in the minor groove (red lines—hydrogen bonds; length [Å]). (**A**) hydration site H2; (**B**) hydration sites H4–H5; (**C**) lack of hydration sites H6–H7.

Compound **11** with the additional benzene ring is oriented towards the bottom of the minor groove, which makes it possible to create three hydration sites H2, H4 and H5 (Figure 9).

The hydration site H2 is present near the amidinium group located between the adenine 17 and guanine 16. The linker of compound **11**, located at the bottom of the DNA minor groove, forms direct hydration sites H4 and H5 with participation of the O3' and O2' atoms of the phosphate group at thymine 8 and thymine 20, respectively. The sulfobenzene substituent also takes part in these interactions.

**Figure 10:**
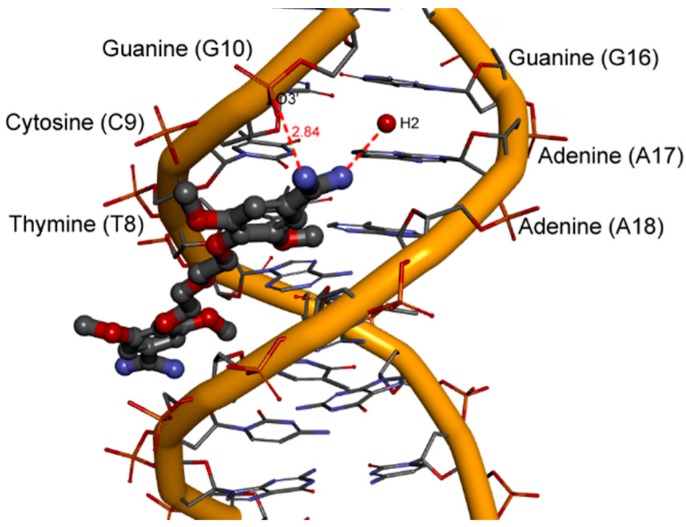
A view of one hydration site for compound **8** in the minor groove (red lines–hydrogen bonds; length [Å]).

For the least active compound **8** bearing four methoxy groups at benzene rings, only one weak interaction through water molecules and the upper amidinium group was observed (Figure 10). The H2 hydration site is located in the proximity of adenine 17 and guanine 16.

The MD studies show that the ligand can form direct and indirect H-bonds with the DNA bases, where water-mediated H-bonds compensate for the lack of curvature of the ligand. The water molecules extend the ligand structure as part of the complex and play an essential role as mediators between the ligand and the DNA surfaces. We can deduce that the most active compounds form more hydration sites than those less active. The additional aromatic ring or the additional methoxy substituent does not allow the formation by water molecules of a continuous array in the minor groove.

## 3. Experimental Section

Pentamidine (**PN**) was purchased as isethionate, from Sigma Aldrich. A series of thirteen compounds **1**–**13** (Table 1) and **TC1**–**TC6** (Table 2) were synthesized in our laboratory [13,34,35]. The lyophilized monomer of dodecanucleotide 5'-(CGCGAATTCGCG)_2_-3' purified using the HPLC method was purchased in Laboratory of DNA Sequencing and Oligonucleotides Synthesis, Institute of Biochemistry and Biophysics, Polish Academy of Sciences, and kept at −20 °C before the experiments. Double-stranded DNA was prepared by heating monomers to 100 °C and a slow cooling to room temperature. This procedure leads to spontaneous binding of complementary DNA strands into double-stranded helical DNA.

### 3.1. DNA Melting Point Measurements (T_m_)

The T_m_s of the oligonucleotide complexes were measured using the UV spectrophotometric method [26]. Absorbances were recorded on a SHIMADZU spectrophotometer (Shimadzu Scientific Instruments, Inc., Columbia, MD, USA), model UV-1601 PC. All absorbance measurements were performed in a buffer system consisting of 2 mM sodium phosphate, 60 mM NaCl, 0.1 mM EDTA with the pH adjusted to 7.0. For absorbance measurements, the buffer systems were scanned at 260 nm in 1 cm quartz cuvettes. The registered absorbance was in the range of 0.5–2.5. The thermal melting experiments were carried out by controlling the temperature of the sample cell with a Julabo (JULABO EH-5) circulating bath while monitoring the absorbance at 260 nm. The temperature of the solution was continuously measured using a DS-1820 (Dallas Semiconductors) digital thermometer (Maxim Integrate Inc., San Jose, CA, USA) working with the accuracy of 0.1 °C in the range of 20–80 °C. The heating rate was about 1 °C/min. Before the measurements the cuvette chamber was purged with nitrogen at 12 °C to avoid condensation. All experiments were performed in triplicate. The results of temperature/absorption experiments were analyzed using the procedures written in Matlab 7.0 (Mathworks) as was shown in [27].

### 3.2. ATP Bioluminescent Assay for Bisamidines

The *in vitro* ATP bioluminescent assay to evaluate the activity of compounds **1**–**13** and **TC1**–**TC6** against *Pneumocystis carinii* was described in [13,36]. The IC_50_ for each bisamidine was calculated using linear regression of the percent decrease in ATP content *vs.* the log drug concentrations (GraphPad Software v2 for Science; GraphPad, San Diego, CA, USA). The IC_50_ values in µM are given in Table 1 and Table 2.

### 3.3. Computational Methods

Docking studies were performed using Discovery Studio 4.0 visual interface [37]. The X-ray crystal structure of the pentamidine bound to the minor groove of DNA dodecamer 5'-(CGCGAATTCGCG)_2_-3' was obtained from the RCSB PDB (ID: 1D64.pdb) [17]. The starting conformations of ligands **1**–**13** and **TC1**–**TC6** were constructed on the basis of X-ray and ^13^C CP/MAS NMR solid-state derived structures of structurally related compounds [13,34,35] to eliminate some subjectivity associated with generating the three dimensional structures. Then the geometries of ligands were constructed by a manual exchange of appropriate atoms in the pentamidine molecule which was located in the minor groove in the crystallographic complexes 1D64.pdb. The geometries of all ligands were optimized using the density functional theory (DFT) with B3LYP/6-311+G (d,p) hybrid functional implemented in the Gaussian 03 program [38]. The so-called ESP-atomic partial charges on the atoms of bisamidines were computed using the Breneman model [39], reproducing the molecular electrostatic potential. Next, the ligands were put into the minor groove adopting the isohelical conformation similar to that of pentamidine. Then the pentamidine molecule was deleted. The geometries of DNA complexes were analyzed using the molecular dynamics method (MD).

### 3.4. Molecular Dynamics Simulations

All molecular dynamics simulations were run using the CHARMm force field [40] implemented in the module of Discovery Studio 4.0. All molecular parameters and atomic charges for nucleic acid were taken from a set of charmm27 force field parameters [41,42]. The DNA complexes were surrounded by a periodic box of water molecules described by the TIP3P potential [29]. The periodic box was extended up to a distance of 10 Å from any solute atom. To each complex in a simulated water box additional salt (NaCl) molecules were added to reach a concentration of ~0.15 M, close to physiological conditions [43] using the Solvation module of Discovery Studio 4.0 (sodium chloride molecules were randomly added to the water box).

All energy minimizations and molecular dynamics simulations were performed using the particle mesh Ewald (PME) method [44] for the correct treatment of electrostatic interactions [45]. Furthermore, a dielectric constant of 1 was used, and atom-based non-bonded interactions were truncated beyond 12 Å using a force shift approach [46], which was proven to accurately represent the long-range electrostatic effects in nucleic acids [47]. The non-bonded lists were maintained for pairs within a distance of 14 Å and updated heuristically whenever an atom had moved more than 1 Å since the last update. All simulations in this study were run with weak harmonic restraints (force constant of 2 kcal/(mol Å^2^)) imposed on the Watson-Crick hydrogen bonds present in the 5' and 3' termini base pairs [48,49,50,51].

The initial minimization process consisted of two cycles of minimization. First, the steepest decent algorithm was applied with 100 steps, and then conjugate gradient algorithm was applied with 10,000 steps to reduce poor intermolecular steric contact (until the RMS gradient of the structure was below 0.01 kcal/(mol Å).

The MD protocol contained a heating step performed for 50 ps with a time step of 1 fs. The system was heated from 50 to 300 K. Prior to the production stage, the system was equilibrated by allowing it to evolve spontaneously for some period of time and integrating the equations of motion until the average temperature and the structure remained stable and the total energy converged. This was facilitated by periodically reassigning velocities appropriate to the desired temperatures, which in this study was 300 K. The total number of steps to perform the dynamics simulation was 500 ps. The Leapfrog Verlet algorithm [52] was used to perform numerical integration of the equations of motion using periodic boundary conditions on an orthorhombic box of TIP3P water. To allow one to keep bonds involving the H atoms at their equilibrium length, the SHAKE algorithm [53] was used. It allows one to use a 2 fs time step for the integration of Newton’s equations. The equilibrated system was taken as the starting structure for the long-term molecular dynamics simulation, called production. The production phase involved the NPT ensemble at a constant pressure while the temperature conditions were maintained via Berendsen algorithms [54] conditions at 300 K. The same conditions as those of the final phase of equilibration were used for the production run of 10 ns, and the coordinates were recorded every 10 ps. Trajectory structures for analysis were saved at 0.2 ps intervals from 10 ns MD simulations. We assumed that hydrogen bonds were present when the donor-acceptor distance was smaller than 3.5 Å.

To examine the stability of the studied complexes in the explicit system, root mean square deviation (RMSD) values were calculated for the pentamidine complex 1D64.pdb and compared with the implicit solvation system where the distance-dependent dielectric constant of ɛ = 4*r_ij_* [55] was used. The evolutions of the structure during the simulation were calculated for the DNA-backbone atoms, DNA-base atoms and the atoms of pentamidine bound in the minor groove of DNA.

### 3.5. Binding Free Energy Calculations

Energy analysis was done applying the MD implementation of the MM-PBSA approach (Molecular Mechanics–Poisson Boltzmann with non-polar Surface Area) [56], based on the MD trajectories obtained using explicit solvent molecules.

We calculated the energy difference between the complex and the unbound ligand and DNA from the following formula:

ΔG_bind_ = G_complex_ − G_DNA_ − G_ligand_ (1)

We used 100 snapshots of the solute sampled regularly from the last ns of the MD trajectories, with the water and counter ions stripped away. This method [57] combines the enthalpy or molecular mechanics energies (E_MM_) that represent the internal energies (bond, angle and dihedral: E_BADH_) along with van der Waals (E_vdW_) and electrostatic interactions (E_elec_), with the solvation free energies (G_solv_) calculated by the finite difference Poisson-Boltzmann (PB) model for polar solvation (G_PB_ or G_polar_) and the non-polar contribution (G_non-polar_) as a function of the solvent-accessible surface area (SASA). All terms were computed with the Discovery Studio 4.0 program. The conformational entropy (*S*), was approximated by translational and rotational components calculated at the molecular mechanics level (Equation (2)).

G = E_MM_ + G_solv_ − TS (2)

The G_polar_ contribution was calculated by applying a cubic lattice with 0.5 Å grid spacing and evaluating all pairwise interactions using an internal dielectric constant of 1 and an outside dielectric of 80. The ΔG_non-polar_ was determined as a function of the SASA estimated using Equation (3)

ΔG_non-polar_ = γSASA + b (3)

where γ b and bare empirical constants of 0.00542 kcal/(mol Å^2^) and 0.92 kcal/mol, respectively, for water.

The selected structures were minimized using conjugate gradients for 10,000 steps after 100 steps of the steepest descents. The Newton-Raphson algorithms were then used for 5000 steps with a distance-dependent dielectric of 1/r^2^ (with r being the distance between two atoms) and a dielectric constant of 4 for the electrostatic interactions until the RMS gradient of the structure was less than 0.001 kcal/(mol Å).

Binding free energies were determined by means of Equation (1) using snapshots from the last ns generated by the single and separate trajectory approach. The coordinates for G_DNA_ and G_ligand_ were extracted from the G_complex_ trajectory.

The interactions between the ligands and DNA dodecamer 5'-(CGCGAATTCGCG)_2_-3' were analyzed for the average conformations generated over the last ns of the production simulation. The average structures were subjected to molecular mechanics minimizations to convergence. We have taken into account the findings presented in the papers [51,58] that the hydrogen bond patterns with the minor groove of the DNA monitored during MD simulations were repeated (*i.e*., they oscillated, and the transition from one type of complex to the other was observed multiple times during simulations).

## 4. Conclusions

The structure-activity analysis for the synthetic pentamidine analogs based on the proposed theoretical protocol and on the experimental anti-*Pneumocystis carinii* activities confirmed that the defined shape of molecules (which make possible the fitting to the DNA minor groove) together with amidinium groups and the N, S atoms present in drug molecules (which are able to form H-bonds with nucleic bases), are needed to enhance the potency of the designed chemotherapeutics. Linear correlations formed between the experimental differences of the melting temperature, ΔT_m_, anti-*P. carinii* activity, log(1/IC_50_) and the theoretical free energy of binding DNA-bisamidine complexes, ΔG_bind_ (ΔT_m_ = −0.139ΔG_bind_ − 1.382; R^2^ = 0.942), and (log(1/IC_50_) = −0.029ΔG_bind_ + 4.757; R^2^ = 0.790) have a high predictive ability. Therefore, these models create the basis to help understand the activity of linear pentamidines and supported the importance of the DNA interaction step in the mechanism of biological activity of those closely related pentamidine analogs. The impact of additional functional groups on anti-*P. carinii* activity can be explained in terms of increased interactions with the DNA minor groove. The results confirmed that in the theoretical analysis of biological models the water molecules should be included in the systems as discrete entities. Water molecules can extend the ligand structures and therefore play an essential role as mediators between the ligands and the DNA surfaces. Pentamidine analogs which were less active did not reveal such favorable interactions with water molecules.

## Supplementary Materials

Supplementary materials can be accessed at: http://www.mdpi.com/1420-3049/20/04/5942/s1.
