# Supplementary material for: Probing the Relationship between Anti-Pneumocystis carinii Activity and DNA Binding of Bisamidines by Molecular Dynamics Simulations
Source: Molecules. 2015 Apr 3;20(4):5942–64. doi: 10.3390/molecules20045942 (PMC6272165; doi:10.3390/molecules20045942)
Supplement: Supplementary file 1 [file molecules-20-05942-s001.pdf]

# Supplementary Materials

## 1. Movements of Drug-DNA Complex During Dynamic Simulations

To check the movements of DNA complexes during molecular dynamic simulations we tested the evolution of the structure of pentamidine and the DNA dodecamer 5'-$(CGCGAATTCGCG)_2$-3' complex in the designed explicit system and compared it with the implicit system using the root-mean-square deviation (RMSD) analysis.

Two solvation systems of the ligand-dodecamer 5'-d$(CGCGAATTCGCG)_2$-3' complex were analyzed during the dynamic simulation procedure: the explicit system with discrete water molecules and the implicit system in which the effect of solvent molecules was simulated using a distance-dependent dielectric constant of $\varepsilon = 4r_{ij}$. The pentamidine-DNA complex was taken as an example to compare both systems. The movement of subjected atoms was monitored through RMSD values. The average RMSD values at 300 K over the course of the last 10 ns were outlined for the trajectory structures of the whole DNA–pentamidine complex. Figure S1A–C, present the atom-positional RMSD values for the DNA backbone, the base pairs and pentamidine atoms as the differences between the trajectory structures and the crystallographic structure of the pentamidine—5'-d$(CGCGAATTCGCG)_2$-3' complex.

As can be seen, the smaller values of RMSD and fluctuations were detected for the explicit system. Figure S1A depicts the RMSD values of the DNA backbone, and it can be observed that DNA conformation close to the crystal structure is preserved with the converged RMSD values of ~1.6 and ~1.8 Å in the explicit and implicit solvent systems, respectively. Figure S1B depicts the RMSD values for the DNA base pairs in the pentamidine complex. The average RMSD values are slightly higher (1.8 for explicit and 1.9 for implicit systems) but the tendency remains to be the same. The smallest RMSD values were observed for pentamidine and their average values oscillated between 1.4 and 1.7 Å, respectively, as shown in Figure S1C.

The small RMSD fluctuations indicated that the DNA - pentamidine complexes reach equilibrium during the simulation run. These results additionally imply that the explicit solvation system is a better choice than the implicit one for the investigations of pentamidine-DNA binding processes.

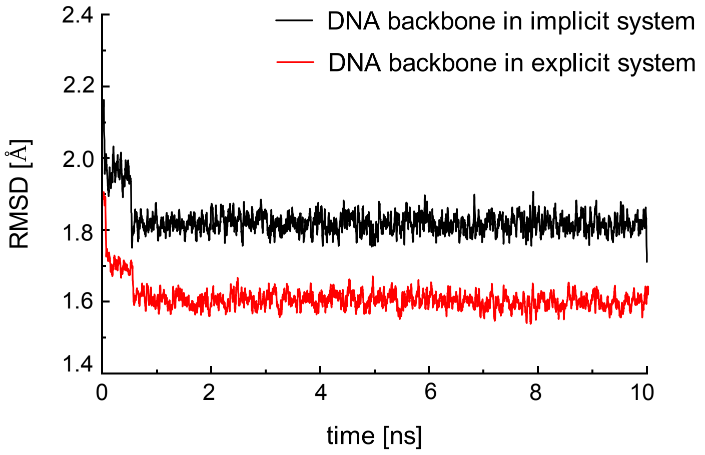


**(A)**

**Figure S1.** *Cont.*

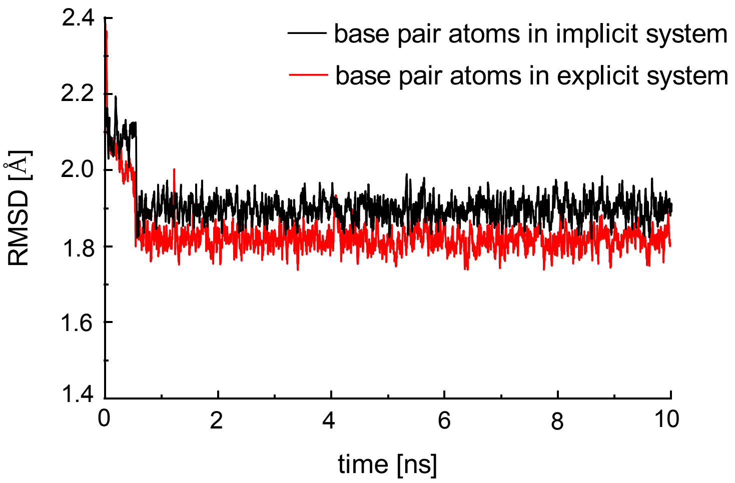


(**B**)

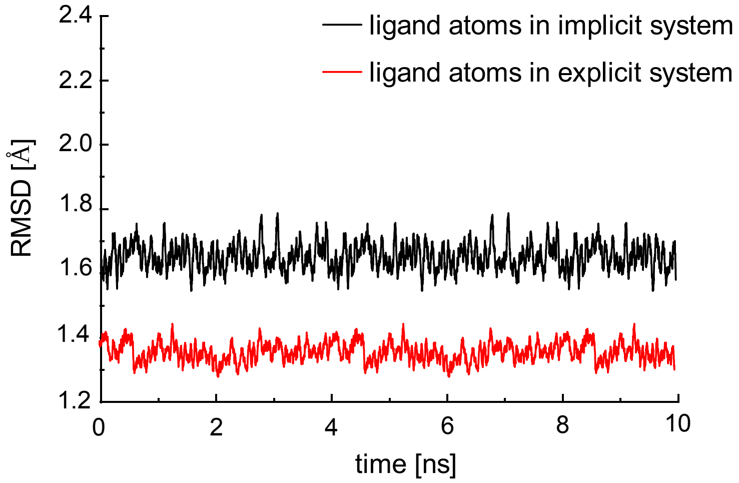


(**C**)

**Figure S1.** Root-mean-square deviation (RMSD) values for the pentamidine-5'-(CGCGAATTCGCG)$_2$-3' dodecamer complex obtained in explicit and implicit solvent approximations during MD simulations: (**A**) for DNA backbone; (**B**) for DNA base pair atoms; (**C**) for pentamidine atoms.

**Table S1.** Each free energy components of Equation (1) estimated for examined subsystems: $G_{DNA+Ligand}$, $G_{DNA}$, $G_{Ligand}$, and $\Delta G_{bind}$ for ligands **PN**, **1–13** and **TC1–TC6**.

| No of Ligand | DNA + ligand G [kcal/mol] | DNA G [kcal/mol] | Ligand G [kcal/mol] | $\Delta G_{bind}$ [kcal/mol] |
|---|---|---|---|---|
| **1** | −3843.672 | | −113.612 | −35.51 |
| **2** | −3853.237 | | −133.107 | −25.58 |
| **3** | −3885.244 | | −170.534 | −20.16 |
| **4** | −3815.577 | | −101.137 | −19.89 |
| **5** | −3878.323 | | −136.483 | −47.29 |
| **6** | −3842.630 | | −121.21 | −26.87 |
| **7** | −3831.299 | | −114.729 | −22.02 |
| **8** | −3855.871 | | −150.661 | −10.66 |
| **9** | −3873.459 | | −151.729 | −27.18 |
| **10** | −3837.275 | −3694.55 | −98.425 | −44.30 |
| **11** | −3810.70 | | −106.50 | −9.65 |
| **12** | −3887.305 | | −165.425 | −27.33 |
| **13** | −3927.74 | | −210.76 | −22.43 |
| **PN** | −3841.78 | | −104.506 | −42.72 |
| **TC1** | −3728.16 | | −107.025 | −26.59 |
| **TC2** | −3698.10 | | −81.42 | −21.13 |
| **TC3** | −3716.93 | | −30.12 | 7.74 |
| **TC4** | −3704.44 | | −97.25 | −12.64 |
| **TC5** | −3768.85 | | −154.85 | −19.15 |
| **TC6** | −3778.52 | | −159.63 | −23.34 |

**Table S2.** Intermolecular interactions of pentamidine analogs **1**–**13** in the minor groove of the 5'-d(CGCGAATTCGCG)$_2$-3' dodecamer.

| Compounds | Ligands Atom (Y) | DNA Atoms (X) | Distance (Y…X) [Å] | Hydration Sites |
|---|---|---|---|---|
| | $N_{Am}\cdots H$ | O4′(C9) | 2.76 | **H1**: O2 (C9), $N_{Am}\cdots H$(**1**) |
| | $N_{Am}\cdots H$ | O3′(A18) | 2.73 | |
| | | O4′(A18) | 3.25 | |
| **1** | $N_{Am}\cdots H$ | - | - | |
| | $N_{Am}\cdots H$ | N3 (A5) | 2.48 | |
| | | O4′(A6) | 2.21 | |
| | O | - | - | **H5**: O3'(T7), O (**1**) |
| | $N_{Am}\cdots H$ | - | - | **H1**: O4'(G10), $N_{Am}\cdots H$(**2**) |
| | $N_{Am}\cdots H$ | O4′(C9) | 2.06 | |
| | | N3 (A17) | 2.51 | |
| | | O4′(A17) | 3.50 | |
| | | O2 (C21) | 2.93 | |
| **2** | $N_{Am}\cdots H$ | - | - | |
| | $N_{Am}\cdots H$ | N3 (A5) | 2.49 | |
| | | O4′(A6) | 2.44 | |
| | | O2 (C21) | 2.57 | |
| | O | - | - | **H5**: O (**2**) |

**Table S2.** *Cont.*

| Compounds | Ligands Atom (Y) | DNA Atoms (X) | Distance (Y…X) [Å] | Hydration Sites |
|---|---|---|---|---|
| **3** | $N_{Am}\cdots H$ | - | - | |
| | $N_{Am}\cdots H$ | O4'(A18) | 3.34 | **H1**: O4'(C9), $N_{Am}\cdots H$(**3**), O3'(C9) |
| | | O3'(A18) | 2.99 | |
| | $N_{Am}\cdots H$ | O3'(C21) | 2.63 | |
| | $N_{Am}\cdots H$ | O2 (C21) | 1.89 | |
| | | O4'(G22) | 2.99 | |
| **4** | $N_{Am}\cdots H$ | - | - | |
| | $N_{Am}\cdots H$ | O4'(A18) | 2.66 | |
| | | N3 (A17) | 3.39 | **H1**: $N_{Am}\cdots H$(**4**), A18, A17 |
| | $N_{Am}\cdots H$ | O3'(A6) | 3.22 | |
| | $N_{Am}\cdots H$ | - | - | |
| **4** | $N_{Am}\cdots H$ | - | - | |
| | $N_{Am}\cdots H$ | O4'(A18) | 2.66 | **H1**: $N_{Am}\cdots H$(**4**), A18, A17 |
| | | N3 (A17) | 3.39 | |
| | $N_{Am}\cdots H$ | O3'(A6) | 3.22 | |
| | $N_{Am}\cdots H$ | - | - | |
| **5** | $N_{Am}\cdots H$ | O4'(C9) | 3.07 | **H1**: O4'(G10), $N_{Am}\cdots H$(**5**), O2 (C9), N3 (G10) |
| | | O3'(C9) | 3.05 | **H2**: $N_{Am}\cdots H$(**5**), N3 (A17), O4'(A17), N2 (G16) |
| | $N_{Am}\cdots H$ | N3 (A18) | 3.26 | |
| | | O4'(A18) | 2.35 | |
| | $N\cdots H$ | - | - | |
| | $N\cdots H$ | - | - | |
| | $N_{Am}\cdots H$ | N3 (A5) | 3.33 | **H4**: $N\cdots H$(**5**), O3'(T8) **H5**: $N\cdots H$(**5**), O4'(T7),O(**5**) **H6**: $N_{Am}\cdots H$(**5**), N2 (G4) |
| | | O4'(A6) | 2.29 | |
| | $N_{Am}\cdots H$ | O4'(C21) | 3.05 | |
| | $N_{Am}\cdots H$ | O3'(C21) | 2.95 | **H7**: $N_{Am}\cdots H$(**5**), O5'(G22), O4'(G22) |

**Table S2.** *Cont.*

| Compounds | Ligands Atom (Y) | DNA Atoms (X) | Distance (Y…X) [Å] | Hydration Sites |
|---|---|---|---|---|
| **6** | $N_{Am}$···H | O4'(C9) | 2.73 | |
| | | O3'(C9) | 2.38 | |
| | | O4'(G10) | 2.95 | |
| | | N3 (A17) | 3.27 | **H2**: $N_{Am}$···H(**6**), A17, G16 |
| | $N_{Am}$···H | O4'(A18) | 2.26 | |
| | | O2 (C9) | 3.03 | |
| | $N_{Am}$···H | - | - | |
| | $N_{Am}$···H | O4'(A6) | 2.84 | |
| | O | - | - | **H4**: O (**6**) |
| | O | - | - | **H5**: O (**6**), O3'(T7) |
| **7** | $N_{Am}$···H | O2 (C9) | 3.35 | |
| | | O4'(A18) | 2.67 | |
| | | N3 (A17) | 3.17 | **H1**: $N_{Am}$···H(**7**), A17, G16 |
| | $N_{Am}$···H | O4'(G10) | 2.98 | |
| | | O3'(C9) | 2.67 | |
| | | O4'(C9) | 3.17 | |
| | $N_{Am}$···H | O3'(C21) | 3.55 | |
| | $N_{Am}$···H | O4'(A6) | 3.25 | |
| **8** | $N_{Am}$···H | O3'(C9) | 2.84 | |
| | $N_{Am}$···H | - | | |
| | $N_{Am}$···H | - | - | **H2**: $N_{Am}$···H(**7**), A17, G16 |
| | $N_{Am}$···H | - | - | |
| **9** | | - | - | |
| | $N_{Am}$···H | - | - | |
| | $N_{Am}$···H | - | - | |
| | $N_{Am}$···H | O3'(C21) | 3.01 | |
| | $N_{Am}$···H | OP2 (G22) | 3.19 | |
| **10** | $N_{Am}$···H | O4' (C9) | 3.00 | **H1**: $N_{Am}$···H(**10**),O4'(G10) |
| | | O3'(G10) | 3.15 | |
| | $N_{Am}$···H | O4'(A18) | 3.04 | **H2**:$N_{Am}$···H(**10**), O4'(A17), N3 (A17) |
| | | | | **H4**: S (**10**), O4'(T20) |
| | S | O2 (T7) | 2.95 | **H5**: S (**10**),O3'(T7), O (**10**) |
| | O | O4'(T19) | 2.97 | |
| | $N_{Am}$···H | O4'(A6) | 2.79 | |
| | $N_{Am}$···H | - | - | **H7**: $N_{Am}$···H(**10**), O3'(C21) |

**Table S2.** *Cont.*

| Compounds | Ligands Atom (Y) | DNA Atoms (X) | Distance (Y…X) [Å] | Hydration Sites |
|---|---|---|---|---|
| **11** | $N_{Am}$···H | O3'(C9) | 2.26 | |
| | $N_{Am}$···H | O4'(G10) | 3.09 | |
| | | O2 (C9) | 2.97 | |
| | | | | **H2**: $N_{Am}$···H(11), A17, G16 |
| | $N_{Am}$···H | O2 (C21) | 3.08 | **H4**: OP2 (T20) |
| | $N_{Am}$···H | O4'(A6) | 3.14 | **H5**: O3'(T8), $O_{sulf}$(11) |
| **12** | $N_{Am}$···H | O2 (C9) | 3.27 | |
| | | O4'(G10) | 2.73 | |
| | $N_{Am}$···H | O4'(A18) | 3.24 | |
| | $N_{Am}$···H | N3 (A5) | 3.20 | **H2**: $N_{Am}$···H(**12**), A17, G16 |
| | | O4'(A6) | 3.19 | |
| | $N_{Am}$···H | - | - | |
| **13** | $N_{Am}$···H | O2 (C9) | 3.18 | |
| | | O4'(G10) | 2.58 | |
| | $N_{Am}$···H | - | - | |
| | $N_{Am}$···H | O4'(A6) | 3.31 | **H2**: $N_{Am}$···H(**13**), A17, G16 |
| | $N_{Am}$···H | - | - | |
